# Diagnostic pitfall in primary cervical gestational choriocarcinoma: a case report

**DOI:** 10.3389/fonc.2025.1608856

**Published:** 2025-08-27

**Authors:** Jiayu Shi, Ping Chen

**Affiliations:** Department of Obstetrics and Gynecology, The First Affiliated Hospital of Henan University of Chinese Medicine, Zhengzhou, China

**Keywords:** cervix, choriocarcinoma, diagnosis, hCG, gestational trophoblastic disease

## Abstract

Choriocarcinoma belongs to a group of highly aggressive malignant gestational trophoblastic neoplasms. Choriocarcinoma is classified into two types based on the origin: a gestational type that arises following a normal or abnormal pregnancy and a non-gestational type resulting from trophoblastic differentiation of germ cells or somatic carcinomas. Primary cervical choriocarcinoma is rare; in women without a recent pregnancy, it is frequently misdiagnosed as other cervical carcinomas, which typically require surgery. Herein, we present a case of primary cervical choriocarcinoma in a 47-year-old woman who presented with irregular vaginal bleeding for five months, with her last pregnancy six years ago. She was initially misdiagnosed with cervical squamous cell carcinoma. Further evaluations, including serum human chorionic gonadotropin (hCG) and comprehensive pathological evaluation, confirmed the diagnosis of gestational choriocarcinoma. Immunohistochemistry showed positive hCG staining, negative p40 and p63 staining, and a high Ki-67 index (60%). Choriocarcinomas of the cervix are uncommon and share similar morphological characteristics with squamous cell carcinoma. Following systemic chemotherapy, hCG levels markedly declined, and the lesion resolved with good response. This case highlights the diagnostic pitfalls associated with primary cervical gestational choriocarcinoma and the importance of clinical correlation, particularly in patients without a recent pregnancy.

## Introduction

Choriocarcinoma is an aggressive type of malignant gestational trophoblastic neoplasm (GTN) that originates from trophoblastic cells and is characterized by a mixture of neoplastic syncytiotrophoblasts, intermediate trophoblasts, and cytotrophoblasts. The incidence of choriocarcinoma varies considerably across regions. In China, choriocarcinoma occurs in 1 out of every 2,882 pregnancies (1); in contrast, in Europe and North America, choriocarcinoma occurs in approximately 1 in 40,000 pregnancies and 1 in 40 cases of hydatidiform moles (1, 2). There are two distinct types of choriocarcinoma: the gestational type, which arises from a normal or abnormal pregnancy (e.g., hydatidiform mole), and the non-gestational type, which arises from trophoblastic differentiation of germ cell tumors or somatic carcinoma (3–5). Abnormal uterine bleeding is the most typical presenting symptom as a result of the endomyometrial invasion by the tumor (6). Choriocarcinoma has a high propensity for distant metastasis, and hemoptysis and abnormal neurological symptoms are initial presentations from lung and cerebral metastases, respectively (1, 7).

Gestational choriocarcinomas most frequently occur in the uterine body, whereas ovary is the most common site in the extrauterine form (8). Primary cervical choriocarcinoma is well documented in the published literature, although rare, with fewer than 200 reported cases (8). When choriocarcinoma arises in the cervix, diagnostic challenges emerge, as cervical choriocarcinoma closely mimics cervical squamous cell carcinoma (SCC). Additionally, differentiating between gestational and non-gestational choriocarcinoma is crucial, as the non-gestational form responds poorly to chemotherapy (3). Here, we present a patient with primary cervical choriocarcinoma and describe the clinical correlation and pathology features, highlighting the diagnostic challenge associated with distinguishing cervical choriocarcinoma and cervical SCC.

## Case presentation

**Figure 1:**
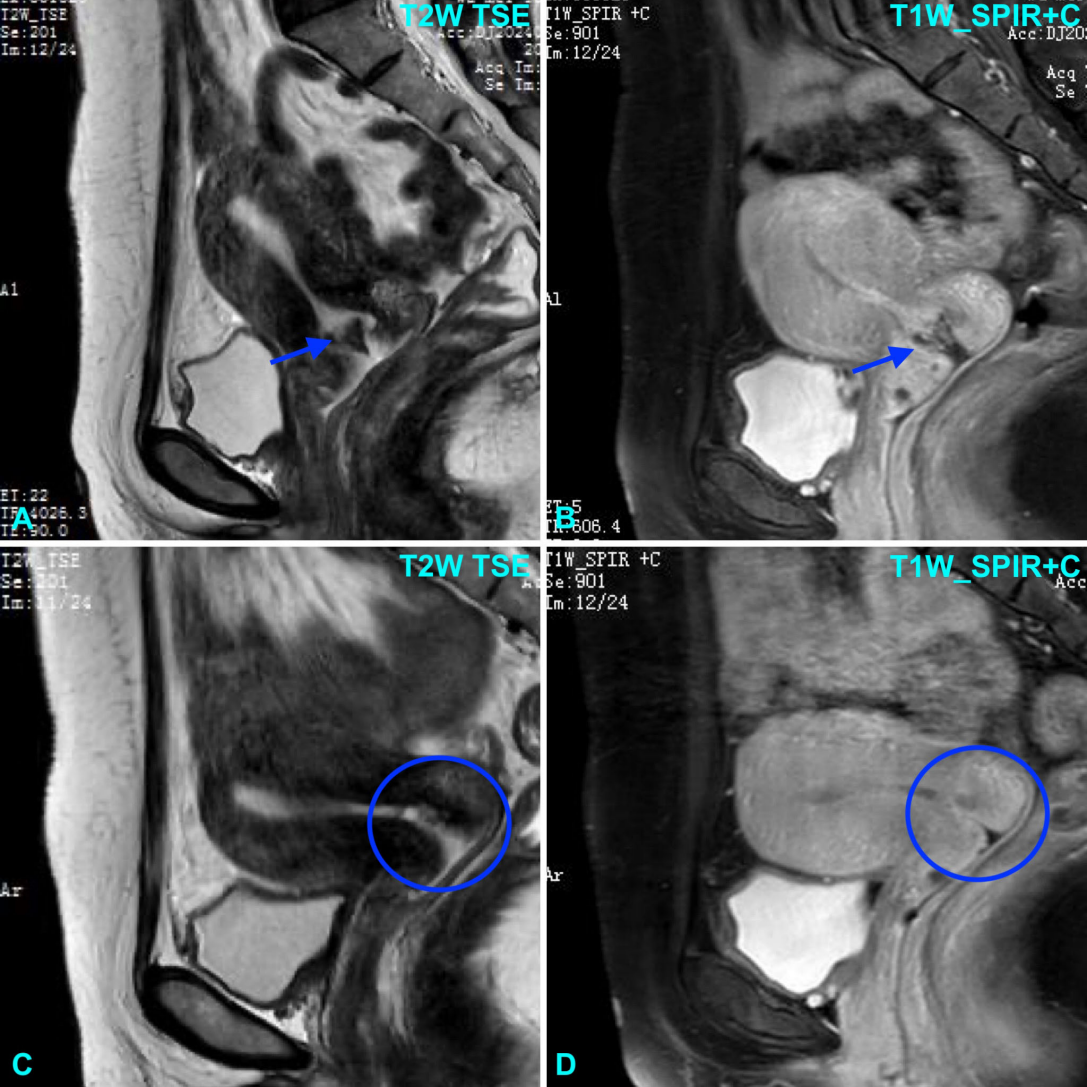
Pelvic MRI shows a lesion centered around the cervix on sagittal T2-weighted Turbo Spin Echo (TSE) image **(A)** and T1-weighted Spectral Presaturation with Inversion Recovery (SPIR) post-contrast image **(B)**. Following chemotherapy, the lesion was no longer visible on sagittal T2-weighted TSE image **(C)** and T1-weighted SPIR post-contrast image **(D)**.

A 47-year-old female with no significant past medical history who presented at a regional community hospital with irregular vaginal bleeding for 5 months. The initial bleeding was outside of her usual menstrual cycle, intermittent, and appears fresh red. The bleeding stopped simultaneously, then recurred after 4 months, and she was referred for further examination. A pelvic ultrasound revealed cervical hypertrophy with multiple cystic areas and a small ovarian cyst. ThinPrep cytology showed no evidence of dysplasia or carcinoma. Two months later, the patient experienced significantly heavier, continuous bleeding. The patient received amoxicillin and unspecified traditional Chinese medicine without resolution of the symptoms. Thus, a cervical biopsy was performed and revealed atypical squamous cells suggestive of SCC. Because of the limited resources at the community hospital, she was transferred to our tertiary medical center for further evaluation. The patient underwent detailed gynecological examination. The vagina was patent with abundant dark red, non-foul-smelling bloody discharge. The cervix appeared enlarged with necrotic and erosion-like changes, measuring approximately 4 cm in diameter, with positive contact bleeding, firm consistency, and a thickened cervical canal. Cervical motion tenderness was present. The uterus was anteverted, normal in size, of medium consistency, mobile, and without significant tenderness. Bimanual examination revealed normal size of the uterosacral or cardinal ligaments, and no obvious abnormalities were noted in the bilateral adnexal regions. Colposcopy revealed a 4 cm necrotic and erosive lesion at the external cervical os. A repeat pelvic ultrasound demonstrated a 4.3 × 3.3 cm heterogeneous lesion with irregular borders spanning the entire cervix. Pelvic non diffuse MRI with and without contrast detected abnormal signals extending from the external os into the endocervical canal (**Figures 1A, B**). Given the location of the lesion, the biopsy findings of atypical cells, and the abnormal imaging results, a diagnosis of cervical SCC was favored. Written informed consent was obtained from the patient, and the study was approved by the ethics committee of the The First Affiliated Hospital of Henan University of Chinese Medicine.

As the patient was of childbearing age, a serum panel for HCG, progesterone, and estradiol was obtained to exclude ectopic pregnancy as a source of bleeding. The results showed a serum hCG level of 7,655 mIU/mL (with a repeat value of 7,346 mIU/mL), a progesterone level of 7.4 ng/mL, and an estradiol level of 107 pg/mL. These findings were perplexing because no radiological evidence of an ectopic pregnancy was observed, and the patient reported regular contraceptive use over the past 6 months with rare exceptions.

The cervical biopsy sample from the outside hospital was re-evaluated. Microscopically, the tumor exhibited an epithelioid appearance characterized by decidualization, marked nuclear pleomorphism, and a hemorrhagic background. Immunohistochemical analysis revealed the following results: hCG (+), p40 (–), CK5/6 (–), p16 (focal +), CEA (–), CK7 (focal +), MUC6 (–), Napsin A (–), p53 showing a wild-type pattern, and a Ki-67 index of approximately 5%. From the morphology and immunohistochemistry profile, a diagnosis of malignant GTN was favored. However, because the Ki-67 index is typically very high in choriocarcinoma, other differential diagnoses of gestational trophoblastic disease could not be excluded.

**Figure 2:**
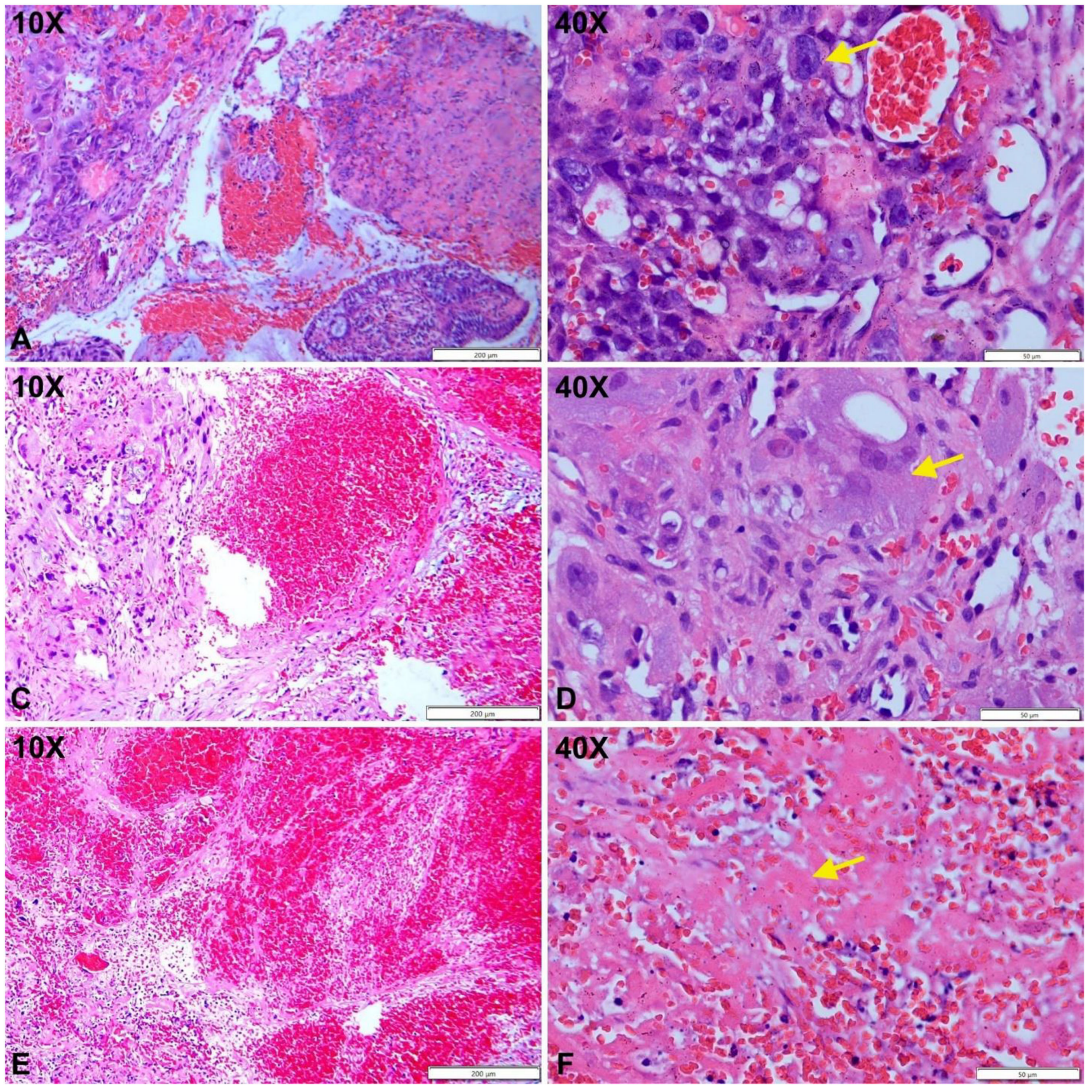
Cytotrophoblast proliferation with cytologic atypia (**A, B**, yellow arrow). Populations of atypical syncytiotrophoblasts are observed (**C, D**, yellow arrow). Areas of hemorrhage with fibrinoid necrosis are noted (**E, F**, yellow arrow).

**Figure 3:**
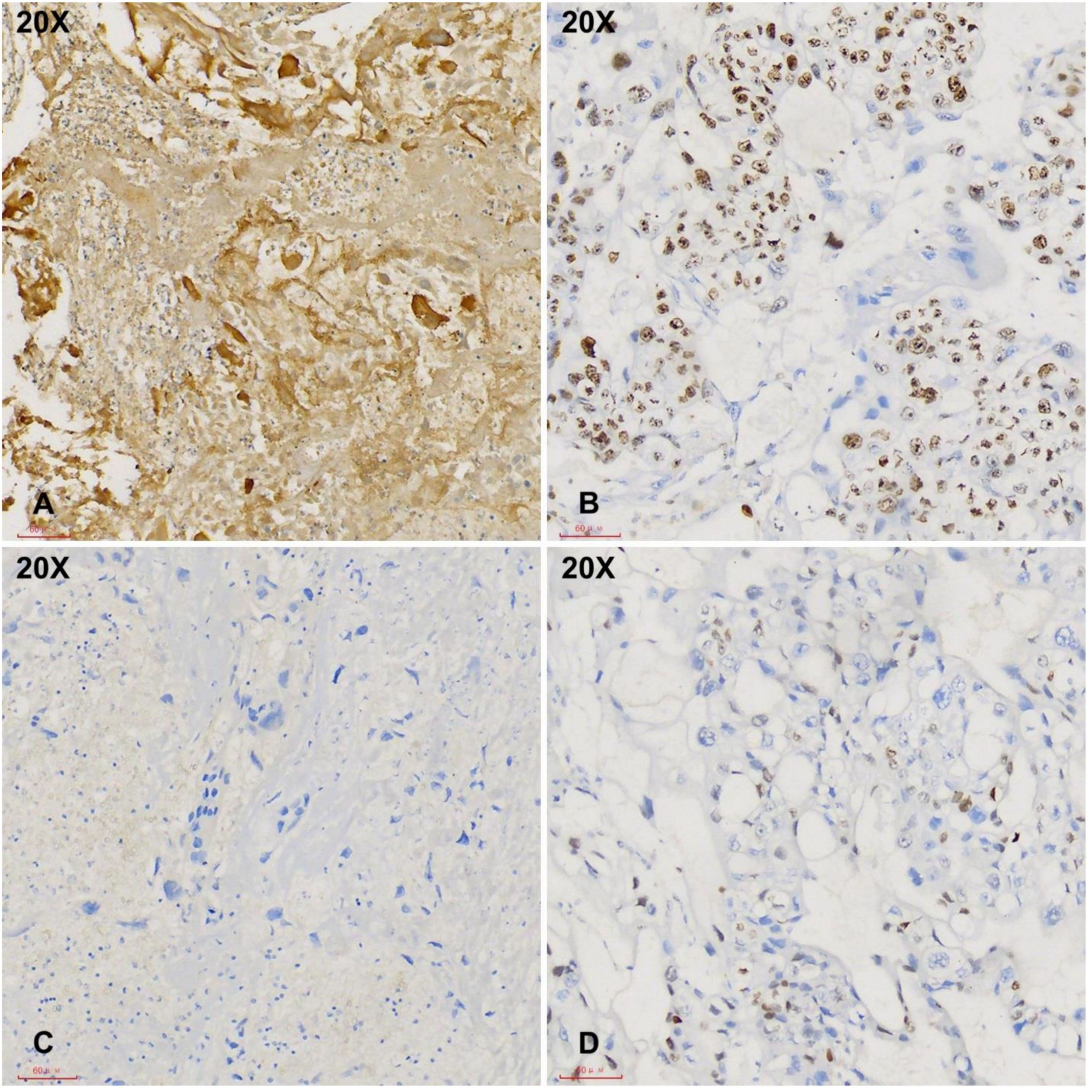
Immunohistochemical studies demonstrate strong hCG positivity in tumor cells with a high Ki-67 index (**A, B**, 20X). The tumor cells are negative for p40 and p63 (**C, D**, 20X).

Immediate hysteroscopy revealed no endometrial abnormalities, and both endometrial biopsy and endocervical curettage were performed. The endocervical curettage specimen demonstrated a similar morphology of pleomorphic cells with squamoid appearance (**Figures 2A–D**). Immunohistochemical analysis showed CK (+), HPL (focal+), hCG (+), CD10 (–), CK5/6 (–), p40 (–), Inhibin A (focal, partial +), MUC4 (focal, partial +), p63 (–), and Ki-67 (60%). The presence of pleomorphic nuclei with abundant pink cytoplasm, hemorrhage, and positivity for hCG, with negative staining for p40 and p63 (**Figures 3A–D**), confirmed the diagnosis of choriocarcinoma. Whole-body CT scan revealed no abnormal lesions suggestive of metastasis.

The patient was over 40 years old with a full-term delivery 6 years prior; her serum HCG levels ranged between 103 and 104 mIU/mL, and the maximum diameter of the tumor was 3–5 cm. Following the 2000 GTN FIGO clinical staging and scoring system, she was diagnosed as stage I with a score of 9, indicating a high-risk subtype (9). The treatment of choriocarcinoma primarily involves systemic chemotherapy, with surgery playing an adjunctive role. The guidelines indicate high-risk GTN is managed with the etoposide, methotrexate, actinomycin D, cyclophosphamide, and vincristine (EMA/CO) chemotherapy regimen. After hysteroscopy and sampling, the patient’s serum hCG level dropped to 506 mIU/mL; following chemotherapy, it decreased to 150.9 mIU/mL, and after a second round of chemotherapy, it dropped to 3.6 mIU/mL (<5 mIU/mL). After completion of three rounds of consolidation chemotherapy, the cervical lesions disappeared (**Figures 1C, D**). The patient’s menstruation has returned to normal, and her hCG levels are monitored on a regular basis. Two additional cycles of EMA/CO chemotherapy regimen were further administered. The patient was followed up every three months and remained disease-free at six months.

## Discussion

Choriocarcinoma is categorized as either gestational or non-gestational, and the difference is crucial in terms of optimal patient care. Gestational choriocarcinoma arises from the malignant transformation of trophoblastic cells following pregnancy. There are approximately 50% of cases associated with hydatidiform moles, 25% with miscarriages, 22.5% seen after a normal pregnancy, and 2.5% following an ectopic pregnancy in women of childbearing age (10–12). Approximately 2–3% of complete moles and less than 1% of partial moles progress to choriocarcinoma (2, 9, 13). The current patient developed gestational choriocarcinoma six years after a full-term delivery when she was 41 years old. The interval between antecedent pregnancy and disease onset is variable, with the longest reported interval of approximately 20 years (14). The pathogenesis of cervical GTN remains unclear. Cervical metastasis may occur despite a primary uterine tumor that spontaneously regresses; alternatively, it may be caused by the malignant transformation of a cervically implanted fetus or trophoblastic cells that migrate to the cervix and transform to malignancy (15, 16). Primary cervical choriocarcinoma may also develop after ectopic pregnancy in cervix and it can have an even normal level of serum hCG (17, 18). The current case represents a true primary cervical choriocarcinoma, as there was no evidence of tumors in the endometrial cavity.

Non-gestational choriocarcinoma results from abnormal differentiation of primordial germ cells during embryonic development. This type of choriocarcinoma is not related to pregnancy and can occur in both men and women, often in conjunction with germ cell tumors such as dysgerminomas and teratomas in the ovary (19, 20). The disease often metastasizes to organs like the lungs, liver, and brain, resulting in a poor prognosis (19). Additionally, a rare but well-established type of non-gestational choriocarcinoma is seen in post-menopausal women, which represents trophoblastic differentiation from somatic malignancy (21, 22).

In patients of reproductive age who present with cervical lesions associated with profuse bleeding, cervical choriocarcinoma is an important differential diagnosis. If serum hCG is not evaluated or unremarkable, pathology examination is the most reliable method to further confirm the diagnosis of choriocarcinoma. Microscopically, choriocarcinoma is characteristic but also shares many features similar to its mimickers. Choriocarcinoma shows marked cytologic atypia, high mitotic activity, and the Ki-67 index is often over 90% (23). The tumor typically appears as an infiltrative mass with extensive hemorrhaging, necrosis, and lymphovascular invasion (24). While the presence of chorionic villi usually rules out the diagnosis of choriocarcinoma, malignant trophoblasts may extend from the villi in term placentas forming intraplacental choriocarcinoma (24, 25). For diagnosis, immunohistochemistry stains such as hCG, HSD3B1, hPL, and inhibin A, are necessary to confirm malignant trophoblasts (5, 26).

In cervical biopsy, SCC is a major diagnostic pitfall, and a patient can be easily misdiagnosed given the morphological and immunohistochemical profile overlap. Both choriocarcinoma and SCC can show significant infiltrative growth, severe nuclear pleomorphism, and b-hCG positivity (27). It is important to note that true squamous differentiation would be positive for one of the two squamous markers, p63 or p40 (28), as long as most cervical SCCs are HPV-mediated and exhibit strong p16 positivity (29). Cervical choriocarcinoma is diagnosed on the basis of diffusely positive hCG, negative p63 and p40, and focal p16. The primary cervical gestational type is further evident by the absence of lesions in the uterine cavity and a very high serum hCG level. The patient has a long latent history (6 years) between the pregnancy and the development of choriocarcinoma. Two possible explanations include an undetected asymptomatic pregnancy with pregnancy loss during the long latent period, and another theory is that retained trophoblastic tissue residing in the cervix following the antecedent pregnancy could remain dormant for years before developing into invasive gestational trophoblastic neoplasms (30).

An important clinical implication of making an accurate diagnosis in this setting is that stage IA cervical squamous cell carcinoma is typically treated with cone biopsy or loop electrosurgical excision procedure, with or without sentinel lymph node mapping, radiation, or extensive surgery, depending on the presence of lymphovascular invasion (31). In contrast, cervical gestational trophoblastic neoplasms are managed primarily with chemotherapy, often combined with surgery (16). The clinical significance of choriocarcinoma developing after a long latent period following an antenatal pregnancy is unclear, particularly regarding whether there is a difference in prognosis or chemotherapy treatment response.

Somatic carcinoma with trophoblastic differentiation is an important differential diagnosis in the current patient, given the associated poor prognosis and different treatment regimens (32). The development of these tumors is not linked to gestational trophoblasts or germ cells (33). Razack et al. reported a case of HPV-associated SCC with trophoblastic differentiation in the cervix and an elevated serum hCG level of 78 mIU/mL (normal <5 mIU/mL). Histologically, the tumor was invasive, showed irregular nuclei membrane, and was devoid of squamous differentiation. The tumor was strongly positive for p16 and GATA3 with a very high Ki-67 index, which strongly mimicked malignant GTNs. In the current patient, the hCG level was over 7000 mIU/mL in two assessments; together with the strong positive hCG detected by immunohistochemical stain, a somatic carcinoma with trophoblastic differentiation can be ruled out. Generally, when immunostain and serum levels of hCG are equivocal, short tandem repeat genotyping is required to confirm the gestational nature with a unique paternal genome that is not present in somatic malignancies (33).

The FIGO staging system categorizes GTN into low and high-risk cases by prognostic scores (stages 7 and 8) determined using a scoring system that takes into account the patient’s age, previous pregnancy, months from pregnancy, hCG level, tumor size, and metastases (9). This patient was at stage I, with a score of 9, indicating a high-risk subtype. Patients with low-risk GTN are treated with single-agent chemotherapy (methotrexate or actinomycin D) (34). Chemotherapy is recommended for 2–3 courses after hCG levels have been normalized. Alternative regimens may be used if the response plateaus (12). GTN that presents as high risk should be treated with the EMA/CO regimen (35). Brain metastases may require additional treatments like cranial radiotherapy, and etoposide and cisplatin are used for induction chemotherapy for extensive metastasis (36, 37). Chemotherapy is the mainstay of GTN treatment, and hysterectomy is usually not required. However, it can reduce chemotherapy cycles in low-risk patients not seeking fertility preservation (38). Surgery is also essential for controlling bleeding or addressing chemotherapy resistance in patients with resistant lesions or severe hemorrhage causing anemia (38, 39).

The current case highlights the diagnostic pitfalls inherent in primary cervical gestational choriocarcinoma, particularly in patients without a recent pregnancy, in which histopathological features overlap with those of more common cervical malignancies. This convergence underscores the need for meticulous clinical correlation to guide appropriate treatment regimens and avoid unnecessary surgical intervention.

## Data Availability

The original contributions presented in the study are included in the article/supplementary material. Further inquiries can be directed to the corresponding author.
